# Regulatory T cells inhibit autoantigen-specific CD4^+^ T cell responses in lupus-prone NZB/W F1 mice

**DOI:** 10.3389/fimmu.2023.1254176

**Published:** 2023-11-10

**Authors:** Stefan Rosenberger, Reinmar Undeutsch, Reza Akbarzadeh, Justus Ohmes, Philipp Enghard, Gabriela Riemekasten, Jens Y. Humrich

**Affiliations:** ^1^ Department of Rheumatology and Clinical Immunology, Charité - University Medicine, Berlin, Germany; ^2^ Helios Dr. Horst Schmidt Kliniken Wiesbaden, Department of General and Visceral Surgery, Wiesbaden, Germany; ^3^ German Rheumatism Research Center (DRFZ), A Leibniz Institute, Berlin, Germany; ^4^ Department of Rheumatology and Clinical Immunology, University of Lübeck, Lübeck, Germany; ^5^ Department of Nephrology and Intensive Care Medicine, Charité - University Medicine, Berlin, Germany

**Keywords:** autoantigen-specific T cells, immune regulation, regulatory T cells, lupus, autoimmunity

## Abstract

**Introduction:**

Progressive loss of regulatory T cell (Treg)-mediated control over autoreactive effector T cells contributes to the development of systemic lupus erythematosus (SLE). Accordingly, we hypothesized that Treg may also have the capacity to suppress the activation of autoreactive CD4^+^ T cells that are considered to drive autoimmunity.

**Methods:**

To investigate whether Treg are involved in the control of autoreactive CD4^+^ T cells, we depleted CD25^+^ Treg cells either *in vivo* or *in vitro*, or combined both approaches before antigen-specific stimulation with the SLE-associated autoantigen SmD1(83-119) in the NZB/W F1 mouse model either after immunization against SmD1(83-119) or during spontaneous disease development. Frequencies of autoantigen-specific CD4^+^ T cells were determined by flow cytometry using the activation marker CD154.

**Results:**

Both *in vitro* and *in vivo* depletion of CD25^+^ Treg, respectively, increased the frequencies of detectable autoantigen-specific CD4^+^ T cells by approximately 50%. Notably, the combined *in vivo* and *in vitro* depletion of CD25^+^ Treg led almost to a doubling in their frequencies. Frequencies of autoantigen-specific CD4^+^ T cells were found to be lower in immunized haploidentical non-autoimmune strains and increased frequencies were detectable in unmanipulated NZB/W F1 mice with active disease. *In vitro* re-addition of CD25^+^ Treg after Treg depletion restored suppression of autoantigen-specific CD4^+^ T cell activation.

**Discussion:**

These results suggest that the activation and expansion of autoantigen-specific CD4^+^ T cells are partly controlled by Treg in murine lupus. Depletion of Treg therefore can be a useful approach to increase the detectability of autoantigen-specific CD4^+^ T cells allowing their detailed characterization including lineage determination and epitope mapping and their sufficient *ex vivo* isolation for cell culture.

## Introduction

The increase in numbers and frequencies of activated and memory-differentiated CD4^+^ T cells in lymphatic organs and inflamed tissues in systemic lupus erythematosus (SLE) suggests that autoreactive CD4^+^ T cells recognizing a distinct panel of autoantigens essentially contribute to immune pathogenesis (1). Among other autoantigens, the C-terminus of the SmD1 protein, the SmD1(83-119) peptide (SmD1p), is considered a key autoantigen in both murine and human SLE (2). T and B cell-driven immune responses against this particular self-peptide can be detected in the majority of SLE patients as well as in mouse models for lupus (3, 4). Despite the well-accepted importance of autoantigen-specific CD4^+^ T cells in lupus, little is known about their characteristics and how their activation and expansion are controlled.

Different techniques have been used to identify autoantigen-specific CD4^+^ T cells to date; however, the reliable detection and isolation of autoantigen-specific CD4^+^ T cells are strongly limited due to the very low prevalence of these cells, which is considered to be lower than 1 per 10^5^ T cells (5). These low frequencies often do not allow the isolation and characterization of autoantigen-specific CD4^+^ T cells. The use haplotype dependent MHCII-peptide multimers to detect antigen-specific CD4+ T cells is still substantially restricted due to the common unavailability of suitable constructs. Other approaches, such as ELISPOT assays for cytokine secretion or flow cytometric intracellular cytokine staining used to detect antigen-specific CD4^+^ T cells do not allow for the isolation of living cells. Recently, CD154, also known as CD40L, has been demonstrated to be a reliable marker for the identification of antigen-specific CD4^+^ T cells (6). The transient expression of CD154 on the surface of CD4^+^ T cells upon antigenic stimulation can be used to assess living antigen-specific CD4^+^ T cells in both human and animal models (6, 7). For the successful isolation of antigen-specific CD4^+^ T cells, investigators have often used foreign antigens. However, the detection of autoantigen-specific CD4^+^ T cells in systemic autoimmune diseases is even more challenging since endogenous mechanisms of self-tolerance, such as the suppression of autoimmune responses by CD4^+^CD25^+^FoxP3^+^ regulatory T cells (Treg), may inhibit their activation in the ubiquitous presence of autoantigens. Meanwhile, there is a general consensus that Treg are indispensable for controlling autoimmunity by keeping autoreactive conventional T cells (Tcon) and other harmful immune cells in check (8). Recent studies indicate that Treg exhibit a higher avidity for autoantigens compared to conventional CD4^+^ T cells (Tcon) (9). Furthermore, it is generally assumed that Treg can prevent the activation of naïve CD4^+^ T cells and their differentiation into effector/memory CD4^+^ T cells. Other studies also suggested a suppressive effect of Treg on already differentiated effector/memory CD4^+^ T cells (10).

SLE is characterized by progressive loss of Treg-mediated control over effector/memory Tcon due to an acquired deficiency of the Treg growth and survival factor IL-2 resulting in an imbalance between Treg and Tcon that advances during the disease course (11–14). Hence, the adoptive transfer of CD4^+^FoxP3^+^CD25^+^ Treg into NZB/W F1 mice with established disease or expansion of endogenous Treg by IL-2 delayed disease progression and increased the survival time (11, 15, 16). By contrast, *in vivo* depletion of CD25^+^ Treg using a depleting anti-CD25 antibody accelerated the development of murine lupus (11). However, in SLE, as a disease with autoreactivity toward several autoantigens, the close interaction between Treg and autoantigen-specific CD4^+^ effector T cells remains to be elucidated. As shown before, immunization of lupus-prone mice against the SmD1p led to a strong disease acceleration (3, 17). Taking this into account, continuous activation and expansion of autoantigen-specific CD4^+^ Tcon may reflect a failure of the corresponding Treg to control their activation adequately. However, the tight control of autoantigen-specific effector CD4^+^ Tcon by Treg cells could also account for the difficulties in reliably identifying these rare cells. Concerning this matter, we could show in a previous work that *in vitro* depletion of CD25^+^ Treg prior to antigen stimulation led to the unmasking of autoreactive CD4^+^ T cell responses against SmD1p in patients with SLE (18). To provide more in-depth evidence for the inhibitory role of Treg in an autoantigen-specific context in lupus, we investigated here whether the *in vitro* and *in vivo* removal of Treg could facilitate the detection and characterization of autoantigen-specific effector/memory CD4^+^ T cells in the NZB/W F1 mouse model for lupus.

## Materials and methods

### Mice

Lupus-prone female New Zealand Black × New Zealand White F1 (NZB/W F1) and non-lupus-prone female Balb/c x New Zealand White F1 (CW F1) mice were obtained from the Federal Institute for Risk Assessment, Berlin, Germany, and the Research Facility for Experimental Medicine of the Charité – University Medicine, Berlin, Germany. The mice were kept in a specific pathogen-free (SPF) environment at the German Rheumatism Research Centre (DRFZ), Berlin, Germany. All experiments were performed according to institutional and federal guidelines (State Office for Health and Social Affairs, Berlin, Germany). NZB/W F1 mice of different disease stages were used: young, clinically healthy mice (8-12 weeks of age, in average 10.4 weeks, no proteinuria); mice at disease onset (5-7 months of age, in average 6.4 months, proteinuria = 30-100 mg/dL); mice with established disease (7-9 months of age, in average 8.8 months, proteinuria = 300-2000 mg/dL). Each mouse was analyzed individually.

### Antigens

The SmD1(83-119) peptide (=SmD1p) (VEPKVKSKKREAVA GRGRGRGRGRGRGRGRGRGGPRR) and a control peptide (= control) with amino acids identical to the SmD1(83–119) peptide, however, with a random sequence of the same amino acids (CREKGRVGRGRPAVGRRGVGRPGRRGSRARGEGKGRK), were synthesized according to a standard procedure. For CD4^+^ T cell epitope mapping 7 overlapping 15-mer peptides derived from SmD1(83-119) [epitope 1 = SmD1(83–97), -epitope 2 = SmD1(85–99), -epitope 3 = SmD1(87–101), -epitope 4 = SmD1(90–104), -epitope 5 = SmD1(93–107), -epitope 6 = SmD1(97–111), and -epitope 7 = SmD1(105–119)] were synthesized by JPT Peptide Technologies GmbH, Berlin/Germany. An 11-mer peptide derived from hen egg lysozyme (HEL (106-116) = NAWVAWRNRCK) was used as control for the 15-mer SmD1 peptides. All peptides were solved in PBS, sterile filtered by 0,2µm filters, and tested negative for the presence of endotoxins.

### Generation of SmD1p-specific CD4^+^ T cells by immunization of NZB/W F1 mice and CW F1 mice

For the *in vivo* generation of SmD1p-specific CD4^+^ T cells, 8-10 weeks old female NZB/W F1 mice or 8-10 weeks old female CW F1 mice were immunized intravenously in the tail vein with 25µg of SmD1(83-119) together with 10µg of lipopolysaccharide (LPS, from E. coli 026:B6*C – Sigma-Aldrich, Taufenkirchen/Germany) as adjuvant diluted in 100µl PBS. After 8 days, a booster immunization was performed subcutaneously in the tail base with 25µg SmD1(83-119) diluted in PBS and emulsified with incomplete Freund’s adjuvant (IFA) (1:1) in a total volume of 50 µl. Another 7 days later, mice were sacrificed and spleen cells were harvested.

### *In vivo* depletion of CD25^+^ Treg

For the *in vivo* depletion of CD25^+^ Treg, 8-10 weeks old female NZB/W F1 or CW F1 mice were injected i.v. with 200µg of an anti-CD25-antibody (rat IgG1, clone PC61.5, DFRZ, Berlin/Germany) one week before the initial immunization. The success of depletion was confirmed by flow cytometry in peripheral blood cells using a non-cross-reactive anti-CD25 antibody (clone 7D4, DRFZ, Berlin/Germany).

### Isolation of splenocytes

Spleens from NZB/W F1 or CW F1 mice were mashed individually through a 70µm cell strainer (BD Biosciences, Heidelberg/Germany), and the cell suspension was washed once with PBS/0,2% BSA. Erythrocytes were eliminated by lysis using a solution containing 0,01M KHCO3, 0,155M NH4Cl, and 0.1mM EDTA at pH 7.5 followed by washing with PBS/0,2% BSA/2mM EDTA.

### *In vitro* depletion of CD25^+^ cells

Erythrocyte-depleted splenocytes were incubated with monoclonal PE-conjugated anti-CD25 antibody (7D4) followed by anti-PE MicroBeads (both Miltenyi Biotec, Bergisch Gladbach, Germany) according to the manufacturer’s instructions. CD25^+^ cells were depleted using the AutoMACS (Miltenyi Biotec, Bergisch Gladbach, Germany). The CD25^-^ fraction showed a purity of over 99% as analyzed by flow cytometry.

### Short-term *in vitro* re-stimulation with antigens

For the detection of antigen-specific CD4^+^ T cells and CD4^+^ T cell epitope mapping, splenocytes from un-depleted, *in vivo* CD25^+^ depleted, *in vitro* CD25^+^ depleted and both *in vivo* and *in vitro* CD25^+^ depleted mice were used. Cells were re-stimulated *in vitro* with SmD1p, 15-mers derived from SmD1p, or with control peptides (randomized peptide, HEL) at a concentration of 20µg/ml for 6h in AIM-V medium (Invitrogen, Darmstadt/Germany) in 96-well plates at a cellular concentration of 1x10^7^ cells/ml. For the comparison between surface and intracellular staining of CD154, different concentrations ranging between 5 µg/ml and 100 µg/ml of SmD1p were used for *in vitro* re-stimulation.

### Staining procedures for the detection of antigen-specific CD4^+^ T cells by flow cytometry

For intracellular staining of CD154^+^ cells, 5µg/ml brefeldin A (Sigma-Aldrich, Hamburg/Germany) was added 2 hours after the addition of antigens to the cell culture. After additional 4 hours of stimulation, cells were fixed in 2% paraformaldehyde for 20 min at room temperature, washed three times, and kept in PBS containing 0,2% BSA and 0,01% sodium azide at 4°C until FACS analyses were performed. Fixed cells were permeabilized in PBS/0,2% BSA containing 0.5% saponin and stained intracellularly using allophycocyanin (APC)-conjugated anti-CD154 antibodies (clone MR1, Miltenyi Biotec, Bergisch Gladbach/Germany). Anti-Fc-γ-receptor antibody (clone 2.4G2, 40µg/ml, DRFZ, Berlin/Germany) was added during the staining procedure to avoid unspecific binding. For surface staining of CD154, unconjugated anti-CD40 antibody (clone 1C10, 10µg/ml, BioLegend, San Diego/USA), anti-Fc-γ-receptor antibody (clone 2.4G2, 40µg/ml, DRFZ, Berlin/Germany) and APC-conjugated anti-CD154 antibody (clone MR1, Miltenyi Biotec, Bergisch Gladbach/Germany) were added to the culture from the beginning. After intracellular staining of CD154 or after the *in vitro* re-stimulation period (6h) for the surface staining of CD154, cells were washed with PBS/0,2% BSA and stained with FITC-conjugated anti-CD4 antibody (clone RM4-5, 5 µg/ml, BD Pharmingen, Heidelberg/Germany) on ice for 15 minutes. Stained cells were analyzed with a FACSCalibur 4-color flow cytometer (BD Biosciences, Heidelberg/Germany).

### Staining procedure for intracellular cytokine detection by flow cytometry

Fixed cells were permeabilized in PBS/0,2% BSA containing 0.5% saponin and stained for intracellular cytokines and surface markers. Anti-Fc-γ-receptor antibody (clone 2.4G2, 40µg/ml, DRFZ, Berlin/Germany) was used to avoid unspecific binding. The following monoclonal antibodies were used for intracellular and surface staining: APC-conjugated anti-CD154 (clone MR1, Miltenyi Biotec, Bergisch Gladbach/Germany), FITC-conjugated anti-IFN-γ (clone XMG1.2, DRFZ, Berlin/Germany), FITC-conjugated anti-TNF-α (clone MP6-XT22, DRFZ, Berlin/Germany), PE-conjugated anti-IL-2 (clone JES6-5H4, DRFZ, Berlin/Germany), PE-conjugated anti-IL-10 (clone JES5-16E3, DRFZ, Berlin/Germany), PE-conjugated anti-IL-17 (clone TC11-18H10, DRFZ, Berlin/Germany), Streptavidin-PerCP (BD Pharmingen, Heidelberg/Germany) and biotin-conjugated anti-CD4 (clone GK1.5, DRFZ, Berlin/Germany). FoxP3-staining was performed using FITC- or PE-conjugated anti-FoxP3 (clone FJK-16s, eBioscience, San Diego/USA) according to the manufacturer’s instructions. Stained cells were analyzed with a FACSCalibur 4-color flow cytometer (BD Biosciences, Heidelberg/Germany).

### Statistical analysis

GraphPad Prism 5 software (Windows version, GraphPad Software Inc., La Jolla/USA) was used for statistical analyses. The two-tailed paired t-test was used to compare differences between SmD1p and control peptide-stimulated samples. Mann-Whitney test was used for the comparison of frequencies of SmD1p-specific CD4^+^ T cells between CW F1 and NZB/W F1 mice and to compare differences in the percentages of FoxP3^+^ and FoxP3^-^ cells among CD4^+^CD25^+^ splenocytes at different disease stages in NZB/W F1 mice. Two-way ANOVA was used to compare differences in the frequencies of SmD1p-specific CD4^+^ T cells between undepleted, *in vitro* depleted, *in vivo* depleted and *in vivo* and *in vitro* CD25^+^ depleted samples and between controls. Two-way ANOVA was also used for the comparisons between surface and intracellular staining of CD154. The statistical tests used for each experiment are indicated in the respective figure legends. P values of < 0.05 were considered statistically significant.

## Results

### Discrimination of autoantigen-specific CD4^+^ T cells by intracellular staining for CD154 is superior to cytokine staining

**Figure 1:**
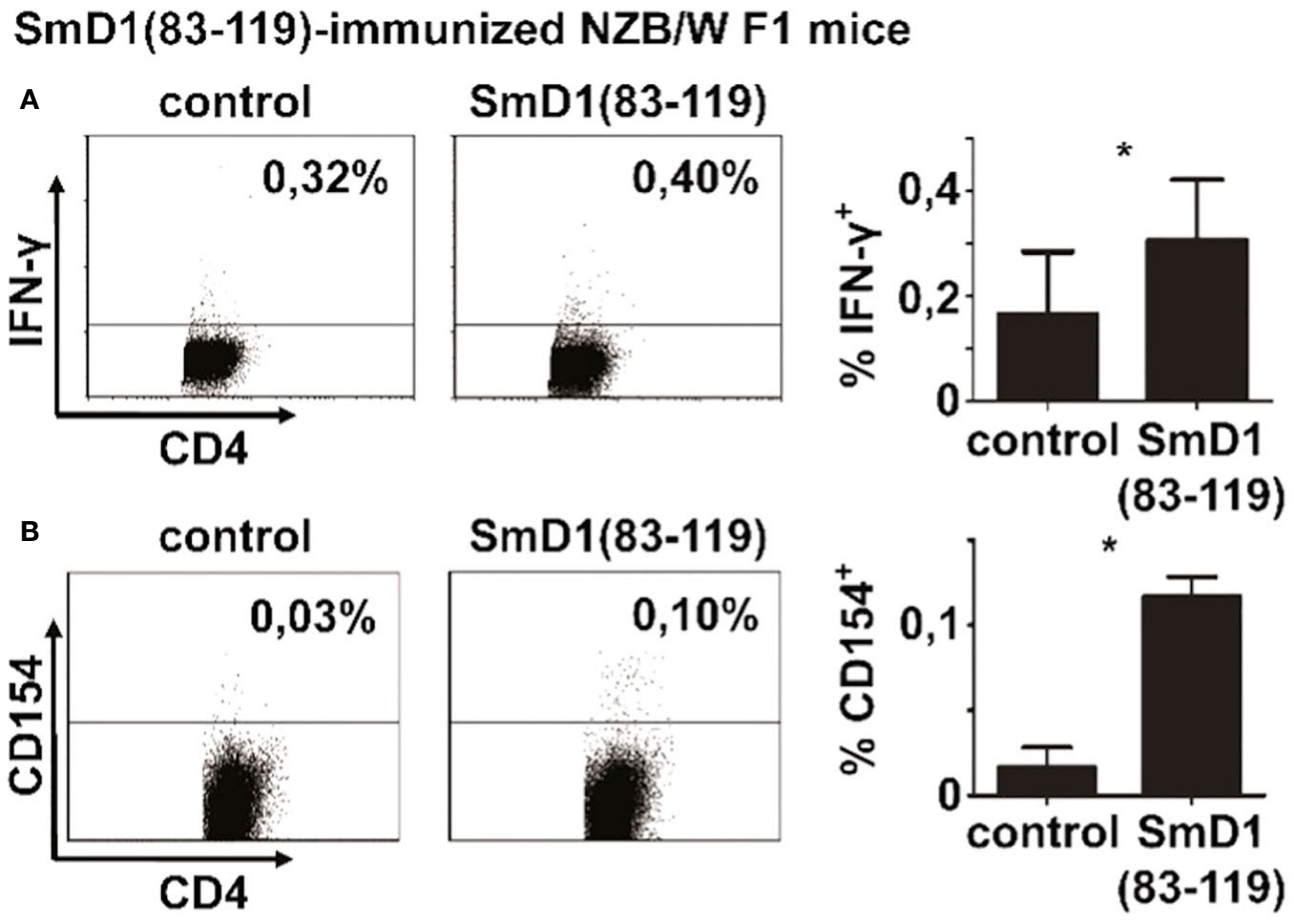
Detectability of autoantigen-specific CD4^+^ T cell responses by different intracellular staining approaches. Splenocytes from SmD1(83-119)-immunized female NZB/W F1 mice at the age of 8-10 weeks were re-stimulated *in vitro* with SmD1(83-119) or a randomized control peptide (= control). Representative dot plots and bar diagrams show the frequencies of SmD1(83-119)-specific CD4^+^ T cells obtained by intracellular cytokine staining for IFN-γ **(A)** and by intracellular staining for CD154 **(B)**. Data in bar diagrams are mean + SD (A, n=6 and B, n=3 per group) derived from 1 or 2 out of 2 independent experiments with 3 individual mice analyzed in each experiment. Two-tailed paired t-test was used (*p<0.05).

Our previous works suggested that the highest frequencies of SmD1p-specific CD4^+^ T cells could be generated by immunization of NZB/W F1 mice against the autoantigen (3). Thus, we primarily used splenocytes from immunized mice for the detection of SmD1p-specific CD4^+^ T cells after antigenic *in vitro* re-stimulation. Firstly, we aimed to compare the yield of detectable SmD1p-specific CD4^+^ T cells obtained by conventional intracellular staining for the Th1 lineage cytokine IFN-γ, the most abundant pro-inflammatory CD4^+^ T cell cytokine in murine lupus (11, 19), with intracellular staining for CD154. Intracellular staining for IFN-γ yielded a mean frequency of 0.32% (range: 0.18-0.40%) of SmD1p-specific IFN-γ^+^ cells among CD4^+^ T cells which were significantly higher than the percentages of CD4^+^IFN-γ^+^ T cells obtained by stimulation with the control peptide (mean 0.16%; p=0.02, SmD1p vs control; **Figure 1A**). However, the unspecific background response obtained by re-stimulation with the control peptide was relatively high representing app. 50% of IFN-γ^+^ cells obtained by re-stimulation with SmD1p. In addition, the staining was diffuse with a high variability in fluorescence intensity and high standard deviations (app. 0.1%) among the individually analyzed mice. Using intracellular staining for CD154 yielded a mean frequency of 0.12% (range: 0.10-0.13%) of SmD1p-specific CD154^+^ cells among CD4^+^ T cells. Although the frequencies of detectable antigen-specific CD4^+^ T cells were lower compared to the IFN-γ^+^ staining, the unspecific background activation was nearly absent when using the CD154 staining approach (mean 0.02% for re-stimulation with control peptide) and standard deviations were low, providing also a higher level of statistical significance for the difference between SmD1p and control peptide stimulated samples (p=0.01, **Figure 1B**). In addition, the discrimination of positively stained CD4^+^ T cells from negatively stained CD4^+^ T cells was superior by using the CD154 staining approach in comparison to conventional cytokine staining. Because of the more reliable detection and superior background discrimination of autoantigen-specific CD4^+^ T cells, we used the CD154 intracellular staining approach for the detection of autoantigen-specific CD4^+^ T cells in our further experiments.

### Depletion of CD25^+^ Treg increases the frequency of detectable autoantigen-specific CD4^+^ T cells

**Figure 2:**
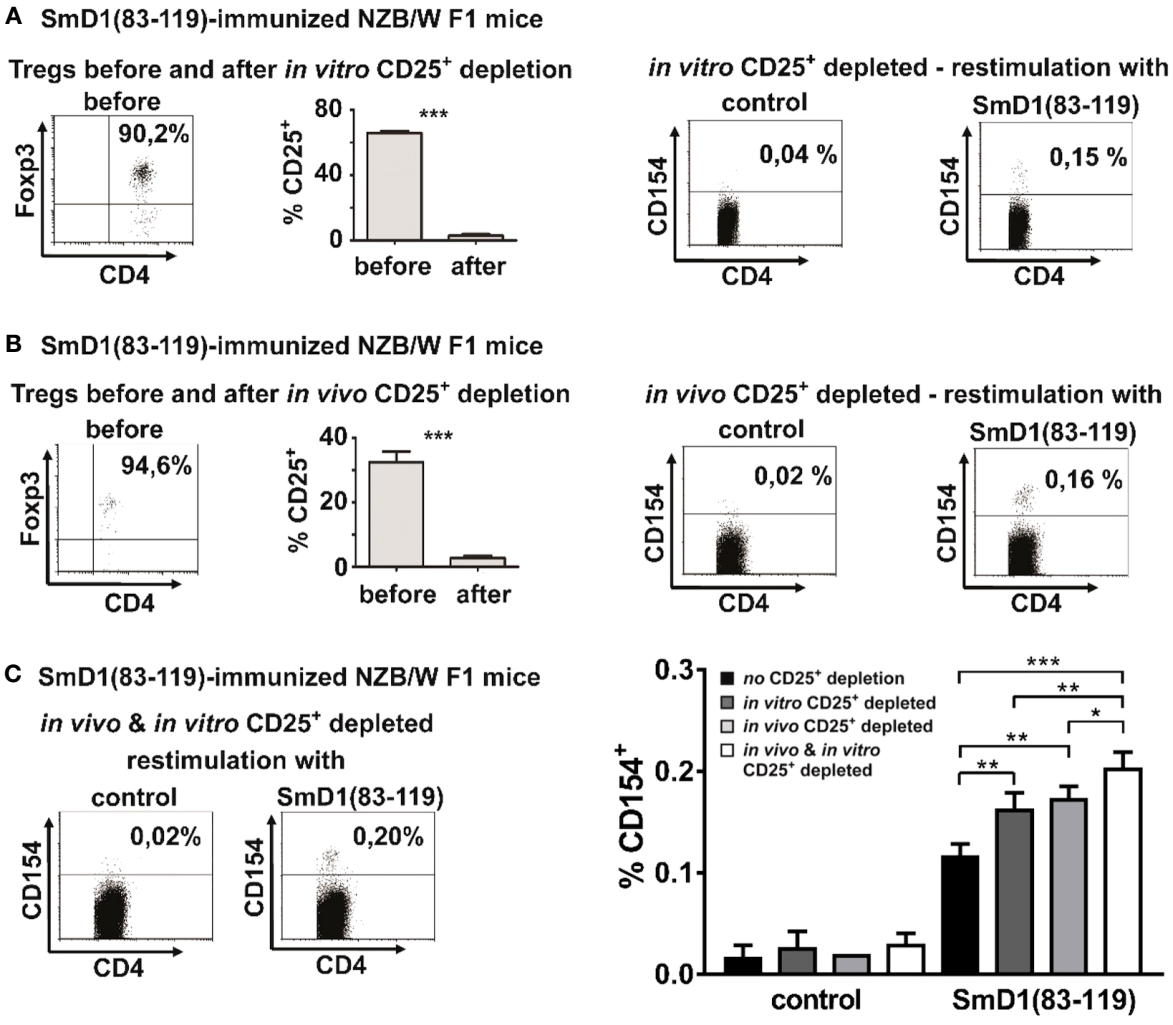
Detection of autoantigen-specific CD4^+^ T cells using different Treg depletion strategies. **(A, B)** NZB/W F1 mice were immunized against SmD1(83-119) and splenocytes were *in vitro* depleted from CD25^+^ cells by MACS **(A)** or NZB/W F1 mice were *in vivo* depleted from CD25^+^ cells by injection of depleting anti-CD25 antibodies (clone PC61) before immunization **(B)**. Dot plots in left panels show the percentage of FoxP3+ cells among gated CD4^+^CD25^+^ T cells before *in vitro* **(A)** or *in vivo* **(B)** depletion of CD25^+^ cells and bar diagrams compare the percentages of CD25^+^ cells among CD4^+^FoxP3^+^ cells before and after depletion of CD25^+^ cells (n=3-5 per group). Representative dot plots on the right side show the percentages of CD154^+^ cells among gated CD4^+^ splenocytes from *in vitro* **(A)** or *in vivo* **(B)** CD25 depleted mice upon *in vitro* re-stimulation with SmD1(83-119) or the control peptide (= control). **(C)** Representative dot plots on the left side show the percentages of CD154^+^ cells among gated CD4^+^ splenocytes from combined *in vitro* and *in vivo* CD25^+^ depleted NZB/W F1 mice upon *in vitro* re-stimulation with SmD1(83-119) or the control peptide (= control). Bar diagram on right side summarizes results obtained by re-stimulation with SmD1(83-119) or the control peptide (= control) using different Treg depletion strategies compared to un-depleted samples. Data in bar diagram are mean + SD (n=3 per group) derived from 1 out of 2 independent experiments with 3 individual mice analyzed in each experiment. Female NZB/W F1 mice at the age of 8-10 weeks were used in all experiments. Two-tailed paired t-test was used for diagrams shown in **Figures 2A, B**, two-way ANOVA was used for bar diagram shown in **Figure 2C** (*p<0.05, **p<0.01, ***p<0.001).

Treg are known for their capability to suppress T cell responses, in particular against autoantigens, in order to efficiently counteract autoimmunity (8). To investigate whether Treg are capable to suppress SmD1p-specific CD4^+^ T cell responses we first depleted CD25^+^ cells *in vitro* from splenocytes of immunized NZB/W F1 mice. Efficient depletion of CD25^+^ cells, of which app. 90% expressed the Treg lineage marker FoxP3 and co-expressed CD4, among the CD4^+^FoxP3^+^ Treg population was confirmed before antigenic re-stimulation (**Figure 2A**, left). Upon re-stimulation with SmD1p, samples depleted of CD25^+^ Treg showed an average increase in the frequency of detectable CD4^+^CD154^+^ T cells by 0.04% compared to un-depleted samples (mean 0.16% vs 0.12%, p=0.0044), whereas the response to the control peptide remained low (mean 0.03%, p<0.0001, SmD1p vs control peptide) comparable to this of un-depleted samples (**Figures 2A, C** right).

To further substantiate this putative inhibitory effect of Treg on antigen-specific T cell responses, we now depleted CD25^+^ Treg *in vivo* by injecting depleting anti-CD25 antibodies before immunization of NZB/W F1 mice against SmD1p (**Figure 2B**, left). *In vivo* depletion of CD25^+^ Treg resulted in an average increase in the frequency of detectable CD4^+^CD154^+^ T cells by 0.05% upon *in vitro* re-stimulation with SmD1p compared to un-depleted samples (mean 0.17% vs 0.12%, p=0.0017) (**Figures 2B, C**, right). This increase in the frequency of SmD1p-specific CD4^+^ T cells was comparable to the increase obtained by the *in vitro* depletion approach (difference not significant). Similar to the *in vitro* depletion experiments, the response to the control peptide remained almost absent (mean 0.02%, p<0.0001; SmD1p vs control peptide; **Figure 2C**, right).

In light of these results, we asked whether the combination of both approaches, the *in vivo* and *in vitro* depletion of CD25^+^ Treg, could have an additive effect on autoantigen-specific CD4^+^ T cell responses. Indeed, the combined depletion approach resulted in the detection of the highest frequencies of CD154^+^ cells among CD4^+^ T cells with a mean frequency of 0.20% (**Figure 2C**), representing an increase of SmD1p-specific CD4^+^ T cells by app. 0.08% compared to un-depleted samples (p=0.0002) and by app. 0.3% and 0.4% compared to *in vitro* (p=0.009) and *in vivo* (p=0.0293) depleted samples, respectively. Again, there was no relevant response to the control peptide (mean 0.03%, p<0.0001; SmD1p vs control peptide; **Figure 2C**) allowing for the reliable discrimination between positive and negative cells.

### Frequencies of autoantigen-specific CD4^+^ T cells are lower in haploidentical non-autoimmune strains and correspond to disease activity in NZB/W F1 mice

**Figure 3:**
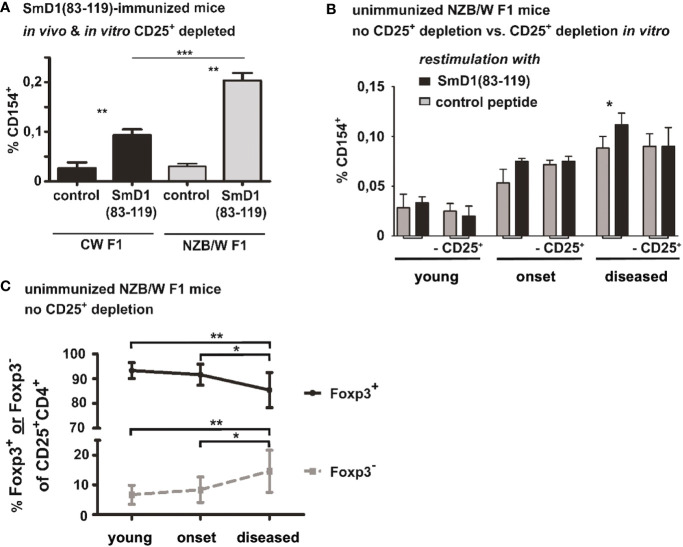
Frequencies of autoantigen-specific CD4^+^ T cells in haploidentical non-autoimmune CW F1 mice and their association with disease activity in NZB/W F1 mice. **(A)** Comparison of SmD1(83-119)-specific CD4^+^ T cell responses in SmD1(83-119)-immunized lupus-prone NZB/W F1 mice versus non-lupus-prone CW F1 mice after combined *in vivo* and *in vitro* depletion of CD25^+^ Treg. Frequencies of CD154^+^ cells among CD4^+^ T cells upon re-stimulation of splenocytes with SmD1(83-119) or the control peptide are shown. Female mice at the age of 8-10 weeks were used for both strains. **(B)** Detection of autoantigen-specific T cell responses during spontaneous disease development in unimmunized NZB/W F1 mice. Frequencies of SmD1(83-119)-specific CD4^+^CD154^+^ T cells from young, healthy mice (no proteinuria, age 8-12 weeks), mice at the onset of disease (proteinuria = 30-100 mg/dL, age 5-7 months), and mice with established disease (proteinuria = 300-2000 mg/dL, age 7-9 months) are shown for un-depleted and *in vitro* CD25^+^ depleted samples upon re-stimulation of splenocytes with SmD1(83-119) or control peptide (= control). **(C)** Percentages of FoxP3^+^ and of FoxP3^-^ cells among gated CD4^+^CD25^+^ splenocytes at different disease stages in unmanipulated NZB/W F1 mice. (n=9 mice per age group) **(A, B)** Shown data are mean + SD (n=3 per group) derived from 1 out of 2 independent experiments with 3 individual mice analyzed in each experiment. Two-tailed paired t-test was used to compare differences between SmD1(83-119) and control peptide stimulated samples **(A, B)**. Mann-Whitney test was used for comparison of frequencies of SmD1(93-119)-specific CD4+ T cells between CW F1 and NZB/W F1 mice **(A)** and to compare differences in the percentages of FoxP3^+^ and FoxP3^-^ cells among CD4^+^CD25^+^ splenocytes at different disease stages in NZB/W F1 mice **(C)** (*p<0.05, **p<0.01, ***p<0.001).

To address whether our observations are specifically associated with susceptibility for lupus, we analyzed for the presence of SmD1p-specific CD4^+^ T cells in the genetically related mouse strain (BALB/c x NZW) F1 (CW F1) which bears the same MHC haplotypes as the NZB/W F1 strain but does not develop lupus-like disease. Immunized CW F1 mice at the same age as NZB/W F1 were subjected to a combined *in vivo* (before immunization) and *in vitro* depletion of CD25^+^ Treg, as described above for the NZB/W F1 mice. Although, SmD1p-specific CD4^+^ T cells according to CD154 expression were detectable above background in CW F1 mice upon *in vitro* re-stimulation (mean 0.09% vs 0.02%, p=0.002, SmD1p vs control peptide; **Figure 3A**) their frequency was more than 2-fold lower compared to the responses observed in Treg depleted NZB/W F1 lupus-prone mice (p=0.002; CW F1 vs NZB/W mice; **Figure 3A**).

To obtain insights into the relevance of SmD1p-specific CD4^+^ T cells and the inhibitory potential of Treg on autoantigen-specific CD4^+^ T cell responses in disease pathogenesis, we examined whether SmD1p-specific CD4^+^ T cells can be also detected and their frequency increased by depletion of CD25^+^ Treg during spontaneous disease development. Accordingly, splenocytes from non-immunized NZB/W F1 mice at different disease stages were *in vitro* depleted from CD25^+^ Treg or left un-depleted before stimulation with SmD1p. In young, clinically healthy NZB/W F1 mice no relevant frequencies of SmD1p-specific CD4^+^CD154^+^ T cells above background levels could be observed, neither in un-depleted nor in depleted samples (**Figure 3B**). Although, frequencies of SmD1p-specific CD4^+^CD154^+^T cells were higher in un-depleted samples of NZB/W F1 mice at the onset of the disease compared to young NZB/W F1 mice, there was no significant difference compared to the control peptide re-stimulation in both un-depleted and depleted samples (**Figure 3B**). However, in mice with established disease there was a significantly higher frequency of SmD1p-specific CD4^+^ T cells in un-depleted samples compared to the stimulation with the control peptide (p<0.05) and the frequency reached similar levels as observed in young mice after immunization with SmD1p, although a much higher level of unspecific background staining for CD154 was evident in the controls suggesting a higher state of constitutive T cell activation in mice with active disease (**Figure 3B**). Unexpectedly, this difference was no more detectable after *in vitro* depletion of CD25^+^ cells as the frequency of SmD1p-specific CD4^+^CD154^+^ T cells dropped to the same level obtained by stimulation with the control peptide. Since CD25 is not exclusively expressed on Treg and is known to be expressed also on activated conventional CD4^+^ T cells (Tcon), and frequencies of CD25^+^ cells among CD4^+^FoxP3^-^ Tcon were shown to be increased in NZB/W F1 mice with active disease (11), the observed loss of SmD1p-specific CD4^+^ T cells after depletion of CD25^+^ cells in diseased mice could be explained by the depletion of the CD4^+^CD25^+^ Tcon population containing also activated SmD1p-specific CD4^+^ T cells. Thus, we analyzed the percentages of FoxP3^+^ and FoxP3^-^ cells within the CD4^+^CD25^+^ T cell population, respectively, at different disease stages. In line with our assumption, the percentages of FoxP3^+^ Treg among CD4^+^CD25^+^ T cells were significantly reduced in diseased NZB/W F1 mice compared to young (p=0.0019) and onset (p=0.04) mice, whereas the percentages of FoxP3^-^ Tcon among CD4^+^CD25^+^ T cells were significantly higher compared to young (p=0.0019) and onset (p=0.04) mice (**Figure 3C**).

### *In vitro* re-addition of CD25^+^ Treg after Treg depletion restores suppression of autoantigen-specific CD4^+^ T cells

**Figure 4:**
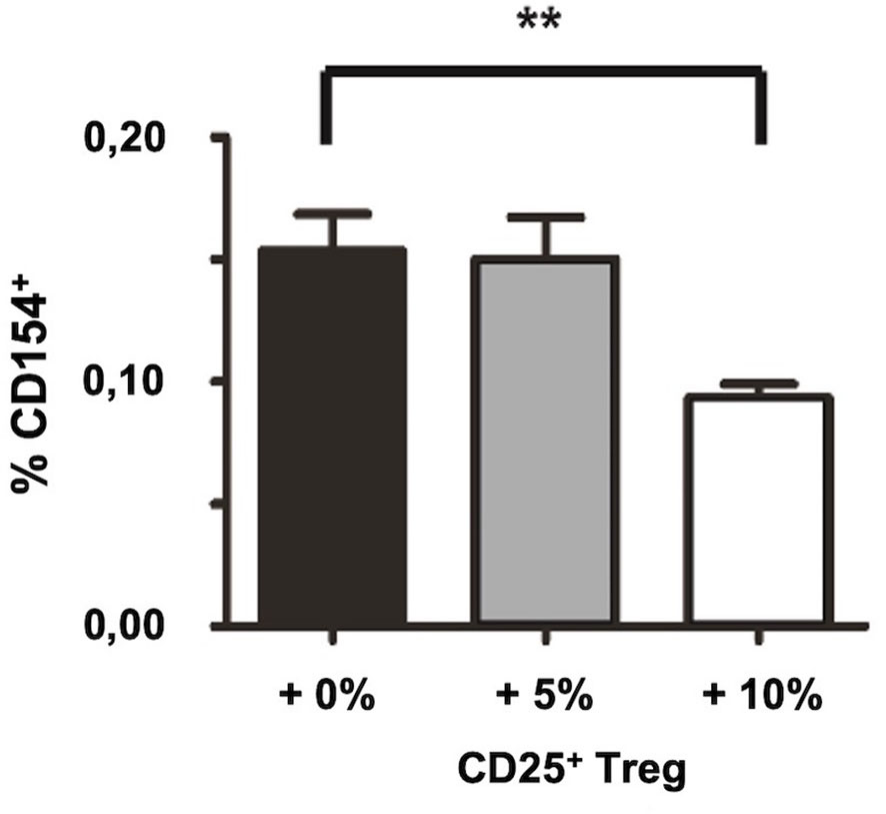
Restoration of suppression of autoantigen-specific CD4^+^ T cell responses by *in vitro* re-addition of CD25^+^ Treg after CD25^+^ Treg depletion. Bar diagram summarizes the frequencies of SmD1(83-119)-specific CD4^+^CD154^+^ T cells upon re-addition of the indicated percentages of CD25^+^ Treg (0, 5, or 10%, respectively) into the cultures before re-stimulation with SmD1(83-119). Female NZB/W F1 mice at the age pf 8-10 weeks were used for all experiments. Shown data are mean + SD (n=3 per group) derived from 1 out of 2 independent experiments with 3 individual mice analyzed in each experiment. Two-tailed paired t-test was used (**p<0.01).

To exclude that the observed increase in the frequency of auto-antigen specific CD4^+^ T cells by depletion of CD25^+^ Treg is biased due to changes in the cellular composition induced by the removal of CD25^+^ cells, we tested whether the *in vitro* re-addition of CD25^+^ Treg after Treg depletion is capable to restore Treg-mediated suppression of autoantigen-specific T cell responses by decreasing the percentage of detectable SmD1p-specific CD4^+^ T cells again. Accordingly, we used splenocytes from *in vivo* and *in vitro* CD25^+^ depleted and immunized NZB/W F1 mice and re-added either none, 5%, or 10% of purified CD25^+^ Treg from unmanipulated, 8-10 weeks old NZB/W F1 mice (total cell number in the cell culture remained the same in all settings) before *in vitro* re-stimulation with SmD1p. Indeed, the re-addition of 10% of purified CD25^+^ Treg resulted in a decrease in the percentages of detectable SmD1p-specific CD4^+^CD154^+^ T cells from a mean of 0.15% to a mean of 0.09% (p=0.01, **Figure 4**), which corresponds to frequencies obtained by stimulation without prior Treg depletion. The re-addition of 5% of purified CD25^+^ Treg, however, was not sufficient to reduce the percentages of SmD1p-specific CD4^+^CD154^+^ T cells.

### Depletion of CD25^+^ Treg facilitates deeper analysis of autoantigen-specific CD4^+^ T cells

**Figure 5:**
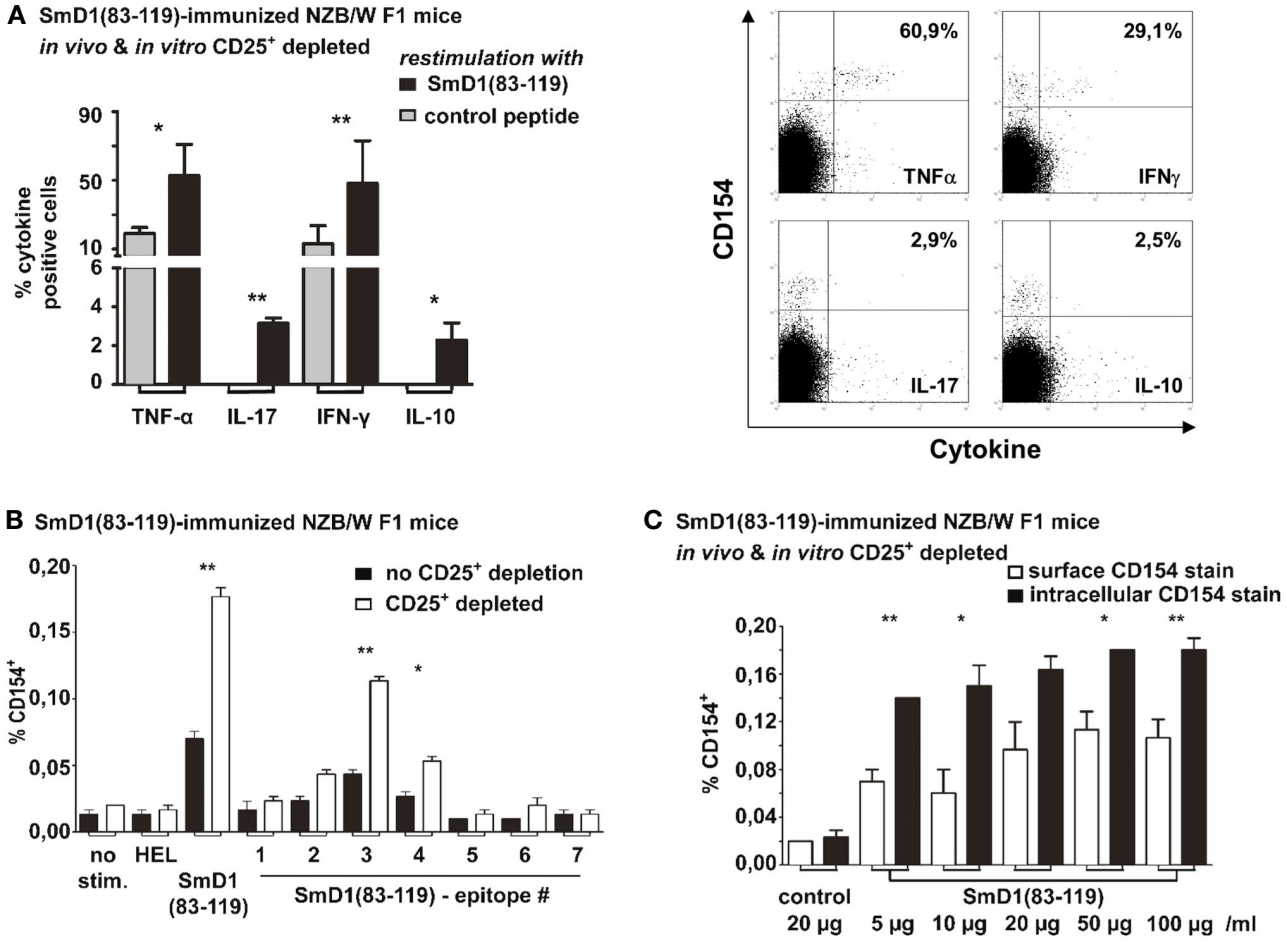
Cytokine profiling and epitope mapping of autoantigen-specific CD4^+^ T cells and comparison of surface and intracellular staining of CD154. Combined *in vivo* and *in vitro* CD25+ Treg depletion was performed in SmD1(83-119) immunized 8-10 weeks old NZB/W F1 mice prior to re-stimulation with SmD1(83-119) or control peptide. **(A)** Percentages and representative dot plots of TNF-α, IL-17, IFN-γ and IL-10 expressing cells among SmD1(83-119)-specific CD4^+^CD154^+^ T cells (n=3-6 per group). **(B)** Frequencies of antigen-specific CD4^+^CD154^+^ T cells obtained by re-stimulation of splenocytes with HEL (11-mer peptide), SmD1(83-119), or different overlapping 15-mer peptide sequences derived from SmD1(83-119) (epitopes 1-7). Shown data are mean + SD (n=3 per group) derived from 1 out of 2 independent experiments with 3 individual mice analyzed in each experiment. **(A, B)** Two-tailed paired t-test was used (*p<0.05, **p<0.01). **(C)** Frequencies of CD154^+^ cells among CD4^+^ T cells are shown for the surface staining of CD154 (on living cells) or intracellular staining of CD154 (in fixed cells) using different concentrations of SmD1(83-119) or a control peptide (= control) for *in vitro* re-stimulation. Shown data are mean +SD (n=3 per group) from one experiment with 3 individual mice. Two-way ANOVA was used (*p<0.05, **p<0.01).

Next, we aimed to delineate the lineage differentiation by determining the cytokine profile of SmD1p-specific CD4^+^CD154^+^ T cells in splenocytes from immunized NZB/W F1 mice that were subjected to the combined Treg depletion approach. We found that SmD1p-specific CD4^+^CD154^+^ T cells produced mainly TNF-α (mean frequency 53%) and IFN-γ (mean frequency 48%), and to a much lesser extent IL-10 and IL-17 (mean frequency < 4% for both), indicating that the majority of SmD1p-specific CD4^+^ T cells belonged to the Th1 lineage (**Figure 5A**).

We also performed fine epitope mapping with 7 different overlapping 15-mer peptide sequences derived from the SmD1p to determine the pre-dominant CD4^+^ T cell epitope. We compared the frequencies of antigen-specific CD4^+^CD154^+^ T cells obtained from immunized NZB/W F1 mice without depletion of CD25^+^ Treg to those from immunized NZB/W F1 mice with combined *in vivo* and *in vitro* depletion. In both experimental settings, re-stimulation with the SmD1p sequence 3 (amino acids 87-101) provided the highest frequencies of epitope-specific CD4^+^CD154^+^ T cells (mean 0.04%, p=0.03 for un-depleted vs control; mean 0.11%, p=0.001 for CD25^+^ depleted vs control; **Figure 5B**). Notably, in the CD25^+^ depleted samples the frequencies of epitope 3-specific CD4^+^CD154^+^ T cells were significantly higher than in the un-depleted samples (p=0.007; **Figure 5B**). Apart from the epitope sequences 2 and 4, which elicited a moderate increase in the frequency of CD4^+^CD154^+^ T cells in CD25^+^ depleted samples, no relevant T cell responses to other peptide sequences were observable (**Figure 5B**). Thus, in NZB/W F1 mice epitope sequence 3 was identified as the dominant CD4^+^ T cell epitope within the entire SmD1p sequence.

One advantage in the detection of (auto)antigen-specific CD4^+^ T cells by CD154 staining and flow cytometry is the possibility to stain CD154 on the surface of living cells, in order to isolate antigen-specific CD4^+^ T cells alive with only little manipulations of these cells. Thus, we compared the frequencies of SmD1p specific CD4+ T cells obtained by surface staining of CD154 with those obtained by the intracellular staining approach by using increasing concentrations of the SmD1p antigen. The surface staining of CD154 on living SmD1p-specific CD4^+^ T cells in general revealed lower frequencies of these cells compared to the intracellular CD154 staining approach for all antigen concentrations tested (**Figure 5C**). However, significantly higher frequencies of SmD1p-specific CD4+ T cells compared to the re-stimulation with the control peptide were evident for all antigen concentrations (p=0.01) and highest frequencies were observed by stimulation with concentrations of 20 µg/ml and 50 µg/ml. The intracellular CD154 staining approach yielded significantly higher frequencies of CD4^+^CD154^+^ T cells than the surface CD154 staining in nearly all concentrations of SmD1p used for re-stimulation (p<0.05 and p<0.01), except for the re-stimulation experiment with the concentration of 20 µg/ml (ns, p=0.06). Similar to what was observed with the surface staining, there was a tendency toward a dose dependency of the SmD1p-specific CD4+ T cell responses (**Figure 5C**).

## Discussion

The detection and characterization of autoantigen-specific CD4^+^ T cells in autoimmune diseases is challenging due to several limitations. First, despite the abundance of autoantigens in autoimmune diseases such as SLE, the frequency of autoantigen-specific CD4^+^ T cells generally is very low and often below the detection limit using flow cytometric approaches (20). Furthermore, the presentation of autoantigens released from dying cells in cell cultures may promote continuous activation of autoantigen-specific CD4^+^ T cells, leading to a refractory state upon further antigenic stimulation (21). Third, CD4^+^ T cell responses to autoantigens can be actively suppressed *in vivo* or *in vitro* by Treg, which mainly recognize autoantigens via their T cell receptors (8).

Here we describe a practical approach to increase the detectability and the yield of these rare cells by flow cytometry through the removal of CD25+ Treg, which opens new possibilities for the deeper classification and enrichment of autoantigen-specific CD4^+^ T cells in a large variety of settings and autoimmune diseases including the identification of immunodominant peptide sequences for the development of suitable MHCII-peptide multimers. In addition, we provide further evidence that Treg are indeed involved in the suppression of specific CD4^+^ T cell responses against autoantigens in murine lupus, as the depletion of CD25^+^ Treg, either *in vitro* before re-stimulation with the autoantigen or *in vivo* before the immunization against the autoantigen, led to an increase in the frequencies of detectable auto-antigen-specific CD4^+^ T cells by approximately 50%. Of note, the combination of both depletion approaches yielded the highest frequencies of detectable auto-antigen-specific CD4^+^ T cells resulting almost in a doubling of their frequencies compared to un-depleted samples. On the other hand, this additive effect could be reversed by the re-addition of Treg to the Treg-depleted cultures, pointing to the restoration of Treg-mediated suppression of autoantigen-specific CD4^+^ T cells and confirming their inhibitory potential. Such an inhibitory effect of CD25^+^ Treg on autoantigen-specific CD4^+^ T cell responses and their unmasking by *in vitro* depletion of CD25^+^ Treg before antigen stimulation was also reported for the detection of SmD1p-specific CD4^+^ T cells in human SLE patients thereby enabling their correlation with disease activity (18).

By using this combined approach, significantly higher frequencies of autoantigen-specific CD4^+^ T cells could be detected in lupus-prone NZB/W F1 mice compared to haploidentical CW F1 mice that are not susceptible to lupus-like disease, suggesting a possible relationship between the generation of SmD1p-specific CD4^+^ T cells and the genetic pre-disposition for lupus. In consideration of our previous work showing a progressively impaired Treg-mediated control over CD4^+^ Tcon activation in NZB/W F1 mice (11), it appears plausible that even in young, clinically healthy lupus-prone mice Treg control autoantigen-specific CD4^+^ T cells to a lesser degree than in a non-lupus-prone mouse strain, which facilitates their escape and clonal expansion. In line with this, we found that frequencies of autoantigen-specific CD4^+^ T cells increased during spontaneous disease progression in unimmunized NZB/W F1 mice, in which the highest frequencies were detectable in mice with established disease, underlying their relevance in disease pathogenesis. This increase occurs in parallel to a progressive impairment of Treg homeostasis due to an acquired deficiency of the Treg growth and survival factor IL-2 promoting an imbalance between Treg and Tcon (11, 16), which also reflects the concomitant and progressive loss of Treg-mediated suppression of Tcon activation.

A practical advantage of using this combined depletion approach together with staining for CD154 is that autoantigen-specific CD4^+^ T cells can be discriminated in a distinct population with higher yields and with low unspecific background staining for further characterization, such as analysis of the cytokine expression profile of autoantigen-specific CD4^+^ T cells. Here we found that IFN-γ and TNF-α were the most abundantly expressed cytokines within the SmD1p-specific CD4^+^ T cell population. The preferential Th1 lineage commitment of SmD1p specific CD4^+^ T cells was somehow expected because of the use of adjuvants during the immunization procedure, which are known to promote Th1 differentiation, such as LPS. Still, these data show that our approach is principally feasible for cytokine profiling and lineage determination of auto-antigen specific T cells, which can be useful for future studies. We also used this approach to identify the predominant CD4^+^ T cell epitope consisting of 15 amino acids derived from the complete SmD1p sequence, confirming previous results with other methods from our group (3). However, as shown here and before, stimulation with the full-length peptide still yielded the highest frequencies of autoantigen-specific CD4^+^ T cells.

There are some limitations of our study. First, to identify relevant and pure frequencies of autoantigen-specific CD4^+^ T cells, immunization with the autoantigen was required, which clearly outperformed the yield obtained from unimmunized mice. However, as immunization with adjuvants strongly influences the cytokine expression pattern and lineage commitment of autoantigen-specific CD4^+^ T cells, the Th1 dominance observed in immunized mice might not necessarily represent the lineage of SmD1p-specific CD4^+^ T cells that are generated spontaneously during disease progression, although a dominant Th1 lineage commitment was also described in previous studies in both human and murine lupus (5, 11, 16, 19, 22). Second, we targeted CD25 expressing cells by using anti-CD25 antibodies for the depletion of Treg in this study which is neither a specific marker for Treg nor sufficient for depleting the entire Treg population, as CD25 is not expressed on a large proportion of CD4^+^FoxP3^+^ Treg, in particular in NZB/W F1 mice in which only app. 30-60% of the CD4^+^FoxP3^+^ Treg population expresses CD25 (11). Therefore, the effects of a complete depletion of the entire CD4^+^FoxP3^+^ Treg population, e.g. by using a transgenic mouse model, on the detection of autoantigen-specific T cell responses remain to be addressed. On the other hand, CD25 is also transiently expressed on recently activated CD4^+^FoxP3^-^ Tcon including autoantigen-specific T cells, which are likewise depleted using anti-CD25-antibodies. Indeed, in unimmunized NZB/W F1 mice with established disease, depletion of CD25^+^ cells could not augment the detectability of autoantigen-specific CD4^+^ T cells. This discrepancy might be best explained by the concomitant removal of activated CD25-expressing CD4^+^ Tcon, containing also SmD1p-specific CD4^+^ T cells, which are known to be increased in established lupus (11). Thus, Treg removal by using depleting anti-CD25 antibodies appears to have its limitations in particular when applied in disease stages with chronic hyperactivity of CD4^+^ Tcon. Third, another possible critical point is that depletion of CD25^+^ cells changes the composition and proportions of the analyzed cell populations and hence the observed increased frequencies of autoantigen-specific CD4^+^CD154^+^ T cells could in part be related to the proportional decrease in the Treg population. Nevertheless, the frequency of depleted FoxP3^+^CD25^+^ Treg is relatively low in NZB/W F1 mice representing app. 5% among total CD4^+^ T cells (11) which does not reasonably explain the increase in autoantigen-specific CD4^+^ T cells from app. 0.1% in un-depleted mice to 0.15 and 0.2% (50 to 100% increase) upon Treg depletion. In addition, the finding that the re-addition of CD25^+^ Treg to Treg-depleted samples could reverse the increase in detectable auto-antigen-specific CD4^+^ T cells, argues against an unspecific proportional effect and rather implies that SmD1p-specific CD4^+^ T cell responses are actively suppressed by Treg. Still, quite high numbers of re-added CD25^+^ Treg (10% of cells in splenocyte culture) were required to elicit this effect, which is likely to result in higher frequencies of CD4^+^FoxP3^+^CD25^+^ among cultured splenocytes than in un-depleted mice. We suggest that such higher numbers of CD25^+^ Treg are required because the re-added CD25^+^ Treg were obtained from non-immunized mice, which might harbor lower frequencies of SmD1p-specific Treg than immunized mice. Another explanation could be that some of the re-added Treg could have changed their functional state during the sorting procedure.

Taken together, this novel combination of methods used for increasing the detectability of autoantigen-specific CD4^+^ T cells provides a feasible approach to studying autoantigen-specific CD4^+^ T cell responses in more depth in both mice and humans allowing their detailed characterization and sufficient *ex vivo* isolation. These results further support the concept that the activation and clonal expansion of autoantigen-specific CD4^+^ T cells are, at least in part, under the control of CD4^+^FoxP3^+^ Treg in lupus.

## Data availability statement

The original contributions presented in the study are included in the article/supplementary material. Further inquiries can be directed to the corresponding author.

## Ethics statement

The animal study was approved by State Office for Health and Social Affairs, Berlin, Germany (LaGeSo). The study was conducted in accordance with the local legislation and institutional requirements.

## Author contributions

SR: Data curation, Formal Analysis, Investigation, Validation, Writing – original draft. RU: Formal Analysis, Investigation, Validation, Writing – original draft. RA: Formal Analysis, Validation, Writing – original draft, Writing – review & editing. JO: Formal Analysis, Writing – original draft, Writing – review & editing. PE: Conceptualization, Data curation, Formal Analysis, Validation, Writing – original draft, Writing – review & editing. GR: Funding acquisition, Supervision, Validation, Writing – original draft, Writing – review & editing. JH: Conceptualization, Data curation, Formal Analysis, Funding acquisition, Supervision, Validation, Writing – original draft, Writing – review & editing.
